# Supplementary material for: Alzheimer’s disease image classification based on enhanced residual attention network
Source: PLoS One. 2025 Jan 27;20(1):e0317376. doi: 10.1371/journal.pone.0317376 (PMC11771898; doi:10.1371/journal.pone.0317376)
Supplement: Attachment — Submitted filename: response to reviewer2.docx [file pone.0317376.s001.docx]

Reviewer #2: The manuscript is technically sound and potentially sound for the scientific community. However, methodological details should be included, and the discussion must be improved before acceptance.
1. Provide a table of existing literature methods with research gaps for clearly mapping.

Sengul Dogan's research paper on EEG signals fills the research gap, and the relevant paper has been cited in this article.

2. The research should address the interpretability of the models' decision-making process.

This paper combines residual learning, attention mechanism, and soft thresholding, to improve the accuracy of image classification.

3. The introduction effectively sets the stage for your research, clearly stating the problem and situating it within the broader field of deep learning and computer vision. It could be enhanced by briefly discussing previous key studies to provide a richer background.

Some key researches have been added in this paper.

4. Some papers can be discussed. For example:
- https://doi.org/10.1007/s11571-024-10104-1
- https://doi.org/10.1007/s11571-022-09859-2
- <https://doi.org/10.1016/j.compbiomed.2021.104828>

The above papers have been discussed and cited in the study.

5. The conclusion successfully encapsulates the study's contributions but could be enhanced by discussing potential future research directions or applications of your findings in a real-world context.

“Future work” has been added in this paper.
6. You should give a proper discussion section and discussions should be increased. Limitations and benefits should be given in bullets.

We have developed the discussion section and explored potential future research directions. Limitations and benefits have been given in the paper.
