# Supplementary material for: Alzheimer’s disease image classification based on enhanced residual attention network
Source: PLoS One. 2025 Jan 27;20(1):e0317376. doi: 10.1371/journal.pone.0317376 (PMC11771898; doi:10.1371/journal.pone.0317376)
Supplement: Attachment — Submitted filename: response to reviewer3.docx [file pone.0317376.s002.docx]

Reviewer #3: The issues are listed in the following:

1. The professional English editing is recommended. The authors should get editing help from someone with full professional proficiency in English.

English major teacher Li Hui helps revising and polishing the entire document.

2. The abstract should highlight the key objectives, methodology, and outcomes clearly. Cite some high quality or relatively new literature, For example, CNN's method introduction can cite "A Novel Centralized Federated Deep Fuzzy Neural Network with Multi-objectives Neural Architecture Search. For Epistatic Detection, DOI: 10.1109 / tfuzz. 2024.3369944 ", The introduction of image segmentation methods can be quoted in "Soft Attention Based DenseNet Model for Parkinson's Disease Classification Using SPECT Images, DOI10.3389 / fnagi. 2022.908143"

The abstract has been revised. Thank you for providing two valuable references, which have been cited in this paper.

3. It is recommended to add an in-depth analysis of the successes and limitations of existing methods and discuss how these compare with your proposed method.

When processing complex medical images, such as MRI images of Alzheimer's disease, existing algorithms are unable to fully capture subtle pathological changes, resulting in suboptimal accuracy. This study compared the performance of ERAN, Attention_Unet, Tiny-VIT, RSN, and MobileNet V3 models in Alzheimer's disease detection tasks, from test accuracy rate and test loss rate.

4. The manuscript needs a concrete justification for the proposed model, particularly the “enhancement” that enables it to work well with the types of images used in the study.

Enhancement is mainly reflected in the powerful effects brought by the added modules. Residual learning, a skip connection network, can reduce the number of layers in the model, optimize model redundancy, and make the model lighter. Attention mechanism enables the model to automatically focus on the lesion area, and improves sensitivity to subtle pathological changes. Soft thresholding effectively filters noise, improves signal-to-noise ratio, and ensures that the model focuses more accurately on features. These viewpoints have been elaborated in the paper.

5. It is suggested to provide more experimental results or case studies to demonstrate the performance of ERAN on different types of medical image data.

We conducted preliminary comparative experiments on ERAN, VGG, and MobileNet models on tumor datasets, and the experimental results are as follows.

Accuracy and loss rate of each model ON TUMOR DATA

| Model | Test Accuracy | Test Loss Rate |
| --- | --- | --- |
| ERAN | 96.62% | 0.2691 |
| VGG | 92.98% | 0.3307 |
| MobileNet | 93.14% | 0.4050 |

6. Provide more charts and visualization tools to clearly show the performance comparison of ERAN and other models on the test set.

Discussion chart about learning rate parameter is added.

7. Conduct an in-depth analysis of the experimental results and explain the reasons for the superior performance of ERAN.

The underlying reasons for the superior performance of ERAN have been analyzed in the experimental results. Please refer to question 4.

8. Strengthen the discussion section by providing an in-depth interpretation of the results and their significance to existing research and clinical practice.

Thank you for your valuable feedback. It has been supplemented in the conclusion part in the paper.

9. Discuss the limitations of the study and propose future research directions.

It has been mentioned briefly in the conclusion part.

10. High-resolution images should be provided to ensure that all details are visible and can be thoroughly examined by readers.

The resolution ratio of the images in the paper has been increased.
