# Supplementary material for: Alzheimer’s disease image classification based on enhanced residual attention network
Source: PLoS One. 2025 Jan 27;20(1):e0317376. doi: 10.1371/journal.pone.0317376 (PMC11771898; doi:10.1371/journal.pone.0317376)
Supplement: Attachment — Submitted filename: response to reviewer120241117.docx [file pone.0317376.s003.docx]

Response to reviewer #1:

1. A detailed analysis of the successes and limitations of existing methods and how they compare with the proposed method

A detailed analysis of the strengths and limitations of existing methods, along with a comparison to the proposed ERAN model, is provided in the introduction section.

2. Providing an in-depth interpretation of the results and their significance to existing research and clinical practice

An in-depth interpretation of the results and their implications for existing research and clinical practice is discussed in the conclusion section.
